# Cellular basis of Alzheimer’s disease

**DOI:** 10.4103/0972-2327.74251

**Published:** 2010-12

**Authors:** Jitin Bali, Saoussen Ben Halima, Boas Felmy, Zoe Goodger, Sebastian Zurbriggen, Lawrence Rajendran

**Affiliations:** Systems and Cell Biology of Neurodegeneration, Psychiatry Research, University of Zürich, 8008 Zurich, Switzerland

**Keywords:** Alzheimer’s disease, amyloid precursor protein, β-secretase, secretase, amyloid, trafficking, endocytosis, exosomes

## Abstract

Alzheimer’s disease (AD) is the most common form of neurodegenerative disease. A characteristic feature of the disease is the presence of amyloid-β (Aβ) which either in its soluble oligomeric form or in the plaque-associated form is causally linked to neurodegeneration. Aβ peptide is liberated from the membrane-spanning -amyloid precursor protein by sequential proteolytic processing employing β- and γ-secretases. All these proteins involved in the production of Aβ peptide are membrane associated and hence, membrane trafficking and cellular compartmentalization play important roles. In this review, we summarize the key cellular events that lead to the progression of AD.

## Alzheimer’s Disease: The Symptoms and the Pathology

Age-related dementia, characterized by cognitive decline and a loss in intellect, affects millions of people worldwide. Alzheimer’s disease (AD) is the most common form of dementia and is described as an irreversible, progressive neurodegenerative disorder. Alois Alzheimer, a German psychiatrist first described this disease in 1906 at a conference in Tubingen, Germany, giving a recount of his 54-year-old female (only identified as Auguste D) patient with presenile dementia. However, AD remained unexplored for many years. In the late 1960s, efforts into AD research rejuvenated and since then have led to the current understanding of the molecular and cellular basis of AD. Because AD is an age-related disorder, the number of people affected by this devastating disorder is expected to increase dramatically in the coming years, particularly in the developed and in developing countries. Approximately 5% of the elderly population aged 65 years or above suffer from AD, and the risk for developing the disease rises sharply with advancing age, doubling approximately every 5 years over the age of 65 and affecting almost one-fourth of elderly people aged 85 years or above. By 2040, an estimated 80 million people will suffer from this disease worldwide.

Often mistook for normal age-related cognitive decline, early symptoms of AD include memory loss and difficulties to retain and recall recently acquired information and hence hard to distinguish. However, the transition from mild to moderate AD is accompanied by an increase in symptom severity, including progressive memory impairment, disordered cognitive function, altered behavior, and a progressive decline in language function. During the final stages of the disease, cognitive performance declines to such a degree that patients require constant help, even for the most basic daily activities. The establishment of a diagnosis is based upon clinical history, physical examination, neuropsychological testing, and neuroimaging. However, a definitive diagnosis is currently still only possible after analysis of brain tissue postmortem.

## Etiology

So, what causes this devastating disease? Although the definitive etiology of the disease is still to be uncovered, several characteristic features of the disease have been investigated. AD is characterized by the selective damage of brain regions responsible for cognition and memory. In the damaged regions of the brain, the dysfunction and death of neurons is associated with cytoskeletal abnormalities and results in a reduction in the levels of synaptic proteins in the regions of the brain in which these neurons terminate. These pathological hallmarks, already described by Alzheimer himself over a century ago, are abnormal intraneuronal cytoskeletal changes, known as neurofibrillary tangles (NFTs), and extracellular protein deposits called amyloid plaques. NFTs are not an exclusive pathological lesion in AD, but can occur in several other neurodegenerative diseases, which are collectively known as tauopathies.

## The Amyloid Pathology

Senile or neuritic plaques are extracellular amyloid deposits found abundantly in the hippocampus and neocortex in AD brain and to some extent in the amygdala. Amyloid deposition within cerebral vessels or cerebral amyloid angiopathy becomes common with advanced age, especially in patients with AD.[1]

A peptide, termed Aβ, forms the core content of the amyloid plaques. Aβ, either in its soluble conformation or in the amyloid plaque-associated form, is causally linked to neurodegeneration. Hence, studying how this amyloid peptide is formed is central in understanding AD pathology. Interestingly, Aβ is generated from a membrane protein called amyloid precursor protein (APP) by two enzymatic activities. The first enzyme that cuts APP is called BACE1 or β-secretase followed by the second enzyme complex dubbed γ-secretase. The latter is composed of several subunits of which the catalytic activity is conferred by multipass membrane proteins called presenilins-1 (PSEN1) or presenilin-2 (PSEN2). Because these secretase activities produce the amyloid peptide, these are attractive therapeutic targets.

## The Genetic Basis of the Disease

Two subtypes of AD have been commonly identified: early-onset (EOAD) and late-onset AD (LOAD), depending on whether disease onset occurs before or after the age of 65 years. EOAD accounts for a small portion (>1 – 2%) of patients suffering from AD, whereas LOAD accounts for the remaining more than 98%. EOAD is most often associated with mutations in the APP gene or in either of the presenilin genes: PSEN1 or PSEN2. These studies fueled AD research by providing cellular and animal models that are routinely used for mechanistic understanding and for screening of drugs. People affected with Down’s syndrome (trisomy 21) display early signs of AD-like behavior, presumably due to gene (APP) dosing effect as APP gene is located in chromosome 21. A prominent genetic risk factor for developing LOAD is the E4 allele of apolipoprotein E. Although there is universal agreement that alterations in the four genes mentioned above can cause AD, various other genes may also play an important role. For example, the gene encoding alpha-2-macroglobulin.[2] In addition, further as yet unidentified genetic factors may have an important function in modifying AD progression. Recently, three new genes, CR1, CLU, and PICALM, were identified to be risk factors for LOAD.[3]

## Biology of Tau and Neurofibrillary Tangles

NFTs contain tau, a microtubule-associated protein as the principal component. Tau plays major roles in the assembly of microtubules, the stabilization of microtubules against dynamic instability, and in bridging these polymers with other cytoskeletal filaments.[4] In AD, NFTs are formed by hyperphosphorylated tau.[5] In normal brain, the equilibrium between phosphorylations and dephosphorylation of tau modulates the stability of the cytoskeleton and consequently axonal morphology. The earliest modification found in Alzheimer brains consists of hyperphosphorylation on tau by action of different protein kinase and phosphatase systems that appear to lead to structural and conformational changes in this protein, thus affecting its binding with tubulin and the capacity to promote microtubule assembly.[6] In human brains, six tau isoforms are produced from a single gene through alternative mRNA splicing which are divided into two groups on the basis of number of microtubule-binding repeats, with three isoforms having three repeats and three isoforms having four repeats each. The presence or absence of N-terminal inserts distinguishes the three isoforms within each group. Similar levels of three and four repeat isoforms are expressed in human brains. Abnormal and excessive phosphorylation of tau by several kinases leads to the release of tau from the microtubules and consequent aggregation in the cytosol. The most relevant protein kinases involved in tau modifications in neurofibrillary degeneration are GSK3β and CDK5.[7] Anti-tau therapies are aimed at inhibiting these kinases or tau aggregation in the cytosol.

## Biology of Amyloid Production

Aβ-peptides are excised from the larger transmembrane APP by the sequential action of β-secretase (BACE1) and γ-secretase.[8] Aβ peptides, released as monomers or oligomers from the cells then form extracellular plaques. Both oligomers and plaques have been demonstrated to have toxic properties including synaptic dysfunction, tau phosphorylation, mitochondrial damage, microglia activation, and neurodegeneration. If Aβ plays such a central role in the pathogenesis of AD, it is imperative to understand the molecular and cellular basis of its production.

## Amyloid Precursor Protein

Amyloid precursor protein (APP) is a type I integral membrane protein, with a large N-terminal extracellular domain and a short C-terminal cytoplasmic domain. It is synthesized in the ER, post-transcriptionally modified in the Golgi, and transported to the cell surface through the secretory pathway. APP is expressed ubiquitously, and Aβ is a normal catabolic product of APP metabolism made in many cells. The APP gene is alternatively spliced to yield isoforms of various lengths; the 751- and 770- (longest) isoforms predominate nonneuronal tissues, whereas the 695-amino acid form (APP695) is by far the most predominant isoform in neurons.[9] APP and APP-like protein have orthologues across nearly all vertebrates and invertebrate animals. The exact role of APP is still not known, but possible roles for APP and its proteolytic products range from axonal transport to transcriptional control and from cell adhesion to apoptosis.[10]

## Processing of Amyloid Precursor Protein

**Figure 1:**
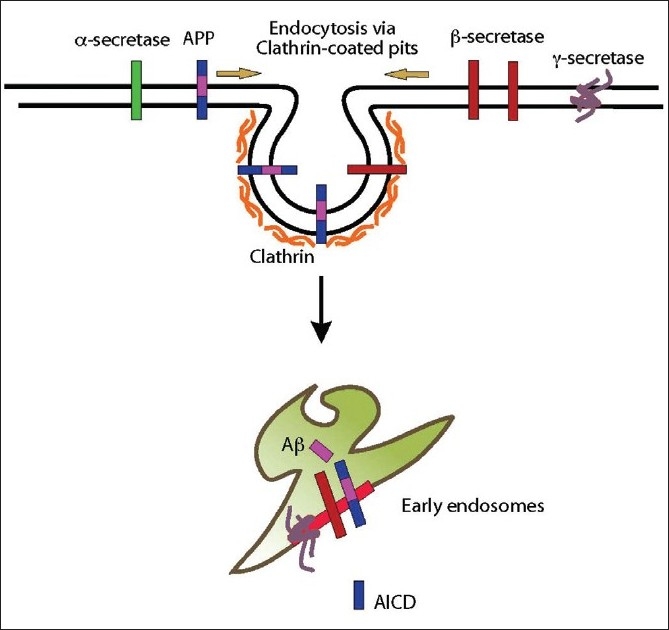
Cell biology of APP processing and amyloid production. APP, and secretases are internalized via clathrin-mediated endocytosis to early endosomes. It is in these endosomes, APP is cleaved by and secretases to produce A peptide and AICD

Though very little is known about its biological function in the cell, very much is known about APP genetics and proteolytic processing with respect to AD. APP can undergo a series of cleavages to generate a wide array of proteolytic products [Figure 1]. APP is first cleaved by either α- or β-secretase at the α- or β-sites, respectively. These two sites lie close to (within ~ 10 – 30 amino acids of) the extracellular side of APP’s transmembrane domain. Cleavage at these sites results in a process known as ‘ectodomain shedding.’[11] These two proteases compete for APP cleavage to give two products: a soluble APP (sAPPα if cleaved by α secretase or sAPPβ if cleaved by β secretase), which is released into the extracellular space and a membrane-anchored C-terminal stub (C83 or C99, for α- and β-secretase cleavage, respectively). It is this stub upon which γ-secretase acts with the peptide bond cleavage occurring within the lipid bilayer. Cleavage of C83 generates the 6-kDa APP intracellular domain (AICD) and releases the N-terminal ~ 3kDa peptide (p3) into the extracellular space. Cleavage of C99 generates AICD and the nefarious Aβ peptide. APP processing by β-secretase followed by γ-secretase leading to the formation of Aβ is called as amyloidogenic pathway. In the alternative pathway of APP processing by α-secretase, no Aβ is produced as α-secretase cleaves APP with an Aβ sequence, thus precluding its formation and so this alternative pathway is termed as nonamyloidogenic pathway.

Aβ peptide is a 4-kDa peptide whose length varies from 38 to 43 amino acids (depending upon the site of γ secretase cleavage), but it is predominantly made up of 40 or 42 amino acids. Aβ is normally secreted by healthy cells throughout life, but its normal physiological function remains unknown. Under physiological conditions, Aβ40 constitutes about 90% of the total amount of Aβ. Of the two major species of Aβ, namely Aβ40 and Aβ42, the later one is more aggregation prone due to addition of two hydrophobic amino acids, thus making Aβ42 the predominant species accumulating in plaques in the AD brain.

## α-Secretase

APP is cleaved by α-secretase in the center of the Aβ domain. Three related metalloproteases of the ADAM (a disintegrin and metalloproteases) family, ADAM-9, ADAM-10, and ADAM-17, also termed as TACE (tumor necrosis factor converting enzyme), appear to exert α-secretase activity. However, the mechanism that regulates α-secretase activity is still unclear. Disintegrin metalloproteases of the ADAM family form a large (at least 40 members in mammals) family of multidomain membrane proteins and are often implicated in ectodomain shedding, either to release growth factors like tumor necrosis factor-alpha or to initiate further intracellular signaling via regulated intramembrane proteolysis. Ectodomain shedding is an additional mechanism whereby cells regulate the protein expression on their cell surface. ADAM-17 is an 824 amino acid polypeptide containing a secretory signal sequence, a disintegrin domain, and a metalloprotease domain.[12] ADAM-9 is composed of 819 amino acids, and northern blot analysis of human and mouse tissues revealed its ubiquitous expression.[13,14] Recent studies from the labs of Bart de Strooper and Paul Saftig have shown that ADAM10 is the main α-secretase that cleaves APP in a nonamyloidogenic manner.

## β-Secretase

β-secretase (BACE1 for β-site APP cleaving enzyme) is a type-I transmembrane aspartyl protease[15] with a luminal active site and is made up of 501 amino acids. It is mainly localized in the endosomes, lysosomes, and the trans-Golgi network. BACE1 has an N-terminal signal sequence and a propeptide domain that are removed post-translationally. It cleaves APP at the N-terminal position of Aβ at the Asp+1 residue of the Aβ sequence. BACE1 is highly expressed in neurons,[15] it can also be expressed in astrocytes under condition of chronic stress. The levels of BACE1 mRNA are highest in pancreas and brain and are significantly lower in most other tissues. Apart from APP, BACE1 is involved in cleavage of neuregulin-1,[16] the protein that has been linked to the pathogenesis of schizophrenia and related psychiatric disorders.[17] β-secretase has maximal activity at acidic pH, because agents that disrupt intracellular pH also inhibit β-secretase activity.[18] Finally, β-secretase is insensitive to pepstatin, an inhibitor of many aspartic proteases.

## γ-Secretase

γ-secretase is a complex responsible for the intramembrane cleavage of the C-terminal stub remained after the cleavage by either α- or β-secretase. γ-secretase cleavage is promiscuous and there is a growing list of non-APP substrates cleaved by this enzyme complex. Of importance, Notch is another widely studied γ-secretase substrate. Interestingly these substrates show little sign of sequence similarity; however, they are all type I transmembrane proteins that require ectodomain shedding as a prerequisite to the γ-secretase cleavage.[19] The promiscuity of -secretase further persists within substrates, as γ-secretase cleavage of APP occurs at a number of sites to generate Aβ peptide of different length.

γ-secretase is a high molecular weight complex comprising of four core components which include Nicastrin, Aph-1, PSEN1 and PSEN2, and Pen 2. Several additional proteins have been proposed to fulfill a modulatory role, including CD147, phospholipase D, and calsenilin.

## Cell Biology of Aβ Production

The core proteins in APP processing which include APP, α-, β-, and γ-secretase are all membrane associated and they are also regulated by their surrounding environment like other membrane-associated proteins. Enzymatic activity of the three secretases is regulated by the pH of the luminal aqueous environment, ionic strength, and nature of the lipids that are found in its immediate neighborhood. Membrane lipids could serve as cofactors, co-structures, or could provide optimal bulk membrane properties that in turn modulate the activity of the enzyme. β-secretase activity is modulated mainly by lipid rafts, which are submembrane assemblies of sphingolipids and cholesterol within the membrane. These lipids form ordered region that segregate from the liquid-disordered matrix of the cellular membrane.[20] A fraction of β-secretase is associated with lipid rafts in cholesterol-dependent manner[21–23] and it is in these raft domains, β-secretase is thought to cleave APP. With the disruption of raft domains by cholesterol depletion, activity of α-secretase (non-raft associated) increased as the fraction of non-raft APP increased and become more available for cleavage by α-secretase.[24] These results led to the interpretation that APP is present in two pools in the membrane, one associated with lipid rafts where β- and -cleavage occurs and other outside of rafts where α-cleavage occurs. β-secretase is active at low pH and needs an acidic compartment for its enzymatic activity, whereas α-secretase is active at the plasma membrane.[25] Cleavage of APP by β-secretase occurs after endocytosis involving the raft-associated proteins, flotillins[26–28] in early endosomes, as these compartments provide low pH required for the activity of β-secretase.[29] Our results and findings from the lab of Christian Hass also showed that γ-secretase cleavage of APP could also occur in early endosomes. In other words, early endosomes are the major cellular sites for Aβ production[29,30] [Figure 1].

Aβ that is generated in the endosomes is released out of the cell, which is necessary for plaque formation and to be released in the cerebrospinal fluid, the latter serves as biomarkers for the progression of AD. An ensuing question is: How does this fairly hydrophobic, intracellularly generated Aβ peptide gets released out of the cell? Our work showed that endosomal Aβ is sorted further to the intraluminal vesicles (ILVs) of multivesicular endosomes.[29,31] When these endosomes fuse with the plasma membrane of the cell, the ILVs are released as exosomes.[32] Exosomes are lipidic vesicles that are approximately 100 nm in diameter and carry selected proteins on them. Of special importance is the prion protein, another amyloid-forming protein that is linked to neurodegeneration.[33]

Aβ-containing exosomes once released or already in the multivesicular endosomes could act as nucleation seeds for plaque formation by recruiting more monomeric Aβ onto them to form fibrils.[34]

## Therapeutic Approaches

The main focus of drug treatment for AD is to improve cognition and slow the progression of symptoms. Current registered treatments cannot cure the disease and an expanding field of research in recent years has been devoted to the development of better treatments, which may eventually lead to a cure. There are currently five drugs approved by the US Food and Drug Administration for the treatment of AD. But all of these drugs are symptomatic drugs. Because the definitive cause is not yet established, it is difficult to design drugs to cure the disease. However, anti-amyloid strategies are on the rise. Because Aβ is causally linked to neurodegeneration observed in AD, anti-Aβ strategies either at inhibiting the production (by inhibiting the secretases) or clearing by immunotherapy have offered some promises in reducing brain Aβ and in improving cognitive functions.[35] Inhibitors of Aβ production are a further growing area of current research interest. These are small compounds that can cross the blood-brain barrier and decrease, but not eliminate, β- or γ-secretase activity. In the case of γ-secretase inhibitors, they should be designed such that they do not significantly interfere with the cleavage of other γ-secretase substrates, including Notch and a number of cell surface receptors, which have vital roles in development, hematopoiesis, and cell adhesion.[1,36] An alternative approach, which may be more promising, is to interfere with the first enzyme, β-secretase. Despite difficulties to generate selective inhibitors, mainly due to the enzyme structure, a number of inhibitors have been developed.[37,38] Interestingly, work from our own group contributed to the identification of novel β-secretase inhibitors by studying the basic cell biology of the processing machinery.[29,39] This finding was crucial as it suggested ways of achieving specificity in inhibiting the Alzheimer’s enzyme.[39]

## Endosomally Targeted β-secretase Inhibitor

If β-cleavage of APP occurs in early endosomes; we reasoned that targeting β-secretase in these endosomes would be a more efficient approach to inhibit the enzyme. The pH of endosomes (pH 4.0 – 5.0) is optimal for β-secretase activity, which explains the requirement of endocytosis for optimal BACE1 function. We examined this hypothesis by testing the efficiency of a membrane-tethered version of an otherwise soluble inhibitor that is now targeted to endosomes via endocytosis. This inhibitor efficiently inhibited β-secretase activity both in cultured cells and *in vitro*. An interesting finding is that although the sterol-linked inhibitors decreased β-cleavage of APP, they also concomitantly increased α-cleavage. This is in agreement with previous findings that inhibition of endocytosis led not only to a decrease in β-cleavage but also to an increase in α-cleavage of APP,[22,24] suggesting that the membrane-anchored inhibitor inhibited the active β-secretase in endosomes.

These data showed that membrane-anchoring dramatically increases the potency of a β-secretase inhibitor. By membrane anchoring of the inhibitor, we achieved two goals which are as follows: (a) the inhibitor became endocytosis-competent and gained access to the endosomal β-secretase; and (b) we reduced the dimensionality of the otherwise soluble inhibitor, thereby enhancing the interaction between the inhibitor and the enzyme.[40] The enhanced potency of the sterol-linked inhibitor conforms to our previous work, where we showed that both lipid environment and the subcellular localization of β-secretase regulate its activity.[22,29] Our data opens up the possibility that this approach can be used to design more effective β-secretase inhibitors for the treatment of AD.[39]

## Outlook

In the production and release of amyloid peptide, subcellular compartmentalization and membrane trafficking play important roles. Designing inhibitors based on the cell biology principles are beginning to pay off. Clearly studying the cellular basis of AD is crucial both for understanding the etiology of the disease and for therapeutic deliberations. Further work is needed to comprehensively understand the role of sorting machinery in other neurodegenerative disorders.
